# Acute exacerbation of chronic osteomyelitis triggered by aggravation of type 2 diabetes mellitus: a case report

**DOI:** 10.1186/s13256-018-1954-y

**Published:** 2019-01-07

**Authors:** Shintaro Irie, Takatoshi Anno, Fumiko Kawasaki, Ryo Shigemoto, Hideaki Kaneto, Kohei Kaku, Niro Okimoto

**Affiliations:** 1 Department of General Internal Medicine 1, Kawasaki Medical School, 2-6-1 Nakasange, Kita-ku, Okayama, 700-8505 Japan; 2 Department of Diabetes, Metabolism and Endocrinology, Kawasaki Medical School, Kurashiki, 701-0192 Japan

**Keywords:** Case report, Type 2 diabetes mellitus, Osteomyelitis, Immunocompromised host

## Abstract

**Background:**

Osteomyelitis is an infection in a bone. Acute osteomyelitis is observed mainly in the long leg bones of children and is usually treated with antibiotics. On the other hand, in adults, subacute or chronic osteomyelitis is more common. Antibiotics therapy is not necessarily effective for chronic osteomyelitis, and sometimes a surgical operation is performed for its remission. Furthermore, in classification of osteomyelitis by cause, type 2 diabetes mellitus is one of most common conditions associated with osteomyelitis. It isCase presentation well known that a variety of complications are induced in patients with type 2 diabetes mellitus due to chronic hyperglycemia, inflammatory reaction, and immunodeficiency, especially when glycemic control is poor.

**Case presentation:**

A 58-year-old Japanese man had acute exacerbation of chronic osteomyelitis triggered by aggravation of type 2 diabetes mellitus. He had acute osteomyelitis in his right lower leg in his babyhood. After this episode, he did not experience any pain in his leg for approximately 50 years; he felt acute pain in his right lower leg at the age of 50 when his glycemic control was very poor. He then started undergoing medical therapy for type 2 diabetes mellitus and, after an improvement in glycemic control, his pain was gradually mitigated. However, he did not take medicine for approximately 8 months at the age of 58. After the interruption, glycemic control became very poor and he felt the similar acute pain again in the same area. After improving glycemic control, his pain was gradually mitigated again as observed at the age of 50.

**Conclusions:**

Here we report a case of chronic osteomyelitis under poorly controlled diabetic conditions. Interestingly, chronic osteomyelitis was observed at the same position where acute osteomyelitis was observed in his babyhood. In addition, chronic osteomyelitis was repeatedly observed, and it seemed that its acute exacerbation was closely associated with aggravation of type 2 diabetes mellitus. We should bear in mind that type 2 diabetes mellitus is one of the major risk factors of osteomyelitis and that acute exacerbation of chronic osteomyelitis could be triggered by a disturbance of glycemic control in patients with type 2 diabetes mellitus.

## Introduction

Osteomyelitis is an infection in a bone. There are many classification systems for osteomyelitis. Traditionally, osteomyelitis was classified with the duration and mechanism of infection [1]. Acute osteomyelitis is observed mainly in the long leg bones in childhood and is usually treated with antibiotics [2]; subacute or chronic osteomyelitis is more common in adulthood. Antibiotics therapy is not necessarily effective for chronic osteomyelitis, and sometimes a surgical operation is performed for its remission [3]. Furthermore, in the classification of osteomyelitis by cause, type 2 diabetes mellitus (T2DM) is one of most common conditions associated with osteomyelitis. Patients with T2DM may develop osteomyelitis in their feet especially when they have a foot ulcer. In such cases, long-term antibiotics therapy is necessary even after the disappearance of its symptom [4].

On the other hand, it is well known that a variety of complications are induced in patients with T2DM due to chronic hyperglycemia, inflammatory reaction, and immunodeficiency. In addition, patients with T2DM have an increased susceptibility to various kinds of infectious diseases such as diabetic foot osteomyelitis, especially when glycemic control is poor [5, 6]. Furthermore, we have to pay much attention even to ulcer and injury, which could become a focus of osteomyelitis [7], because diabetic foot osteomyelitis could develop and invade from an ulcer or injury to the skin.

Here we report a case of chronic osteomyelitis under poorly controlled diabetic conditions. Interestingly, chronic osteomyelitis was observed at the same position where acute osteomyelitis was observed in his babyhood. In addition, chronic osteomyelitis was repeatedly observed, and it seemed that its acute exacerbation was closely associated with aggravation of T2DM. These data suggest that we should always pay attention to acute exacerbation of chronic osteomyelitis in patients with poorly controlled T2DM and acute osteomyelitis as a past history. And we should pay attention to acute osteomyelitis in babyhood; otherwise, we might unexpectedly experience a case with acute exacerbation of chronic osteomyelitis in patients with poorly controlled T2DM.

## Case presentation

A 58-year-old Japanese man with an 8-year history of T2DM had a symptom of pain in his right lower leg and visited the emergency room in Kawasaki Medical School. Previously, he had felt the same pain in the same region when his blood glucose was very high and thereby he was diagnosed as having T2DM 8 years before.

**Fig. 1:**
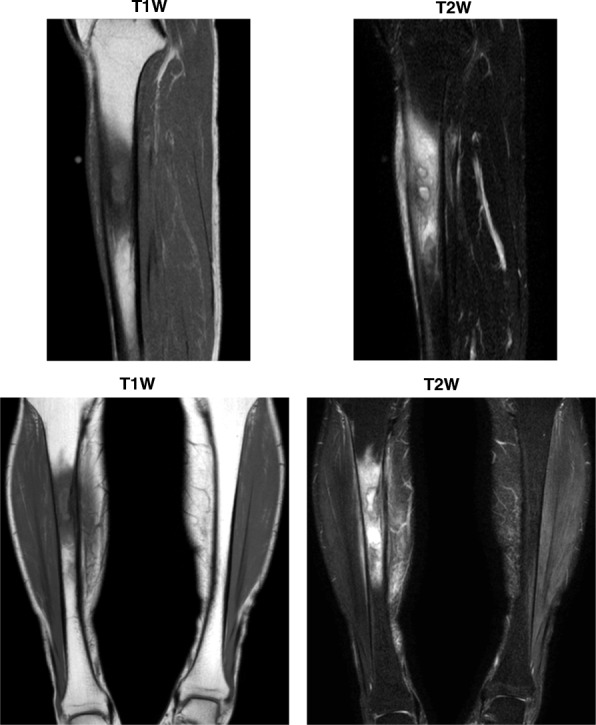
Magnetic resonance imaging showed an abscess and inflammatory change in right lower leg. At the onset of type 2 diabetes mellitus, axial T1-weighted image showed a slightly lower intensity, and axial T2-weighted image showed a markedly higher intensity (*upper panels*). Magnetic resonance imaging showed a spreading of the abscess and inflammatory change in right lower leg after the interruption of therapy at the age of 58 (*lower panels*). *T1W* T1-weighted image, *T2W* T2-weighted image

He had acute osteomyelitis in his right lower leg when he was a baby. Except for this, he had no past history. He had no remarkable family history. He was a barber; he smoked tobacco (pack-years = 0.75 pack/day × 40 years) and he drank alcohol every day. After the episode of acute osteomyelitis when he was a baby, there was no problem in his legs until he had general fatigue and felt pain in his right lower leg at the age of 50. He visited the emergency room. His vital signs were as follows: heart rate 76 beats/minute, blood pressure 116/70 mmHg, and body temperature 36.4 °C. He had a symptom of slight local swelling and heat sensation in the same area with pain in his right lower leg, but there were no findings in physical and neurological examinations. In addition, there was no ulcer or injury on his skin surface. Laboratory data (Table 1) were as follows: white blood cell count, 7400/μL (neutrophil 64.7%); C-reactive protein (CRP), 2.50 mg/dl; plasma glucose, 382 mg/dL; hemoglobin A1c (HbA1c), 11.7%. He was diagnosed as having T2DM, but he had no diabetic complications. Magnetic resonance imaging (MRI) of his lower limbs showed an abscess and inflammatory change in his right lower leg (Fig. 1). An axial T1-weighted (T1W) image of his right lower leg showed a slightly lower intensity, and an axial T2-weighted (T2W) image showed a markedly higher intensity (Fig. 1, upper panels). Based on these findings, we made a diagnosis of acute exacerbation of chronic osteomyelitis and T2DM. We thought that it would be better to hospitalize him and start administering antibiotics via a drip, but he did not agree to the hospitalization. Therefore, as an alternative, we started 300 mg/day of cefcapene pivoxil hydrochloride hydrate and insulin therapy (18 units of aspart) on an out-patient basis. After starting insulin therapy, his blood glucose level gradually decreased, and his leg pain was also gradually mitigated. Finally, his leg pain disappeared 2 weeks later. His CRP became within normal range, and 3 months later the focus in his right lower leg was markedly reduced on MRI. In addition, the focus was not detected in ultrasonography of the right tibia site. Just in case, however, we continued antibiotics therapy for 4 months. Since his glycemic control was improved 2 months later, we stopped insulin therapy and started orally administered anti-diabetic drugs.

**Table 1:** Laboratory data on the onset of type 2 diabetes mellitus at age of 50 and after interruption of diabetic therapy at age of 58

	Onset of T2DM	After interruption
Peripheral blood (reference range)		
Red blood cell count (410–540 × 10^4^/μL)	491	476
Hemoglobin (13.0–16.5 g/dL)	15.9	15.9
White blood cell count (3500–9500/μL)	7400	6680
Neutrophil (52.0–80.0%)	64.7	63.4
Lymphocyte (20.0–40.0%)	27.6	27.2
Platelet (15.0–35.0 × 10^4^/μL)	30.1	25.1
Blood biochemistry (reference range)		
C-reactive protein (< 0.30)	2.50	0.32
Procalcitonin (0.00–0.05)	N/A	0.03
ESR (1–7 mm/hour)	N/A	31
Plasma glucose level (70–110 mg/dL)	382	652
HbA1c (4.9–6.0%)	11.7	6.9
Glycoalbumin (12.4–16.3%)	N/A	46.1
AST (10–35 U/L)	31	39
ALT (7–42 U/L)	27	50
γ-GTP (5–60 U/L)	40	51
LDH (120–240 U/L)	188	172
ALP (110–360 U/L)	326	313
Creatinine (0.60–1.10 mg/dL)	0.57	0.54
BUN (8–22 mg/dL)	12.3	13
Amylase (42–118 U/L)	57	54
Sodium (137–146 mEq/L)	136	131
Potassium (3.6–5.0 mEq/L)	4.5	4.4
Chloride (101–110 mEq/L)	101	96

*ALP* alkaline phosphatase, *ALT* alanine aminotransferase, *AST* aspartate aminotransferase, *BUN* blood urea nitrogen, *ESR* erythrocyte sedimentation rate, *γ-GTP* γ-glutamyl transpeptidase, *HbA1c* hemoglobin A1c, *LDH* lactate dehydrogenase, *N/A* not applicable, *T2DM* type 2 diabetes mellitus

He was then followed up as an out-patient with T2DM for approximately 8 years. The medication at that time was 1000 mg/day of metformin, 25 mg/day of alogliptin, 15 mg/day of pioglitazone, and 50 mg/day of ipragliflozin. However, he did not take the medicine for approximately 8 months on his own judgement at the age of 58. After his interruption of therapy for 8 months, he felt the same pain in the same right lower leg again. He immediately visited our hospital. He had symptoms of slight local swelling and heat sensation together with pain in the same area in his right lower leg, but again there were no findings in physical and neurological examinations at this time. His vital signs were as follows: heart rate 99 beats/minute, blood pressure 130/70 mmHg, and body temperature 37.0 °C. He had a symptom of slight local swelling and heat sensation in the same area with pain in his right lower leg, but there was no ulcer or injury on his skin surface. Laboratory data were as follows: white blood cell count, 6680/μL (neutrophil 63.4%); CRP, 0.32 mg/dl; erythrocyte sedimentation rate (ESR), 31 mm/hour; plasma glucose, 652 mg/dL; HbA1c, 6.9%; glycoalbumin 46.1%. Other laboratory data were as follows: red blood cell, 476 × 10^4^/μL; hemoglobin (Hb), 15.9 g/dL; platelet, 25.1/μL; total protein (TP), 7.6 g/dL; albumin (Alb), 4.4 g/dL. Liver and renal function were within normal range as follows: aspartate aminotransferase (AST), 39 U/L; alanine aminotransferase (ALT), 50 U/L; γ-glutamyl transpeptidase (γ-GTP), 51 U/L; lactate dehydrogenase (LDH), 172 U/L; creatinine (Cre), 0.54 mg/dL; blood urea nitrogen (BUN), 13 mg/dL; Na, 131 mEq/L; K, 4.4 mEq/L; Cl, 96 mEq/L. Pathogenic bacteria were not detected. He had no diabetic complications, probably because his glycemic control was relatively good before the interruption of therapy. His leg MRI showed a spreading of the abscess and inflammatory change in his right lower leg (Fig. 1, lower panels). We hospitalized him in our institution but he did not agree to undergo surgery for remission. Therefore, we started 3.0 g/day of sulbactam sodium/ampicillin sodium and insulin therapy (24 units of aspart and 20 units of glargine). After starting insulin therapy, his blood glucose level gradually decreased, and his leg pain was also gradually mitigated. Local swelling and heat sensation disappeared approximately 5 days later. Finally, his leg pain disappeared approximately 2 weeks later, and he was discharged from our hospital. Just in case, however, we continued antibiotics therapy (450 mg/day of rifampicin and 4 g/day of trimethoprim) for approximately 2 months. After a total of 3-month antibiotics therapy during hospitalization and after discharge, we stopped antibiotics therapy. He was then followed up for approximately 6 months, and his leg MRI showed a reduction of the abscess and inflammatory change in his right lower leg. He had no symptoms and/or problems, and his inflammation markers remained within normal levels for at least 6 months.

## Discussion

Here we report a case of acute exacerbation of chronic osteomyelitis. This patient suffered from acute osteomyelitis in his right lower leg when he was a baby. He had not experienced any pain in the leg for almost 50 years, but he felt acute pain in his right lower leg at the age of 50 when his glycemic control was very poor. Therefore, we think that he had acute exacerbation of chronic osteomyelitis. In addition, it seemed that his repeated symptom of pain in his right lower leg was closely associated with bad control of T2DM. In fact, he had pain in his right lower leg when glycemic control became very poor at the age of 58 as well. Therefore, we finally made a diagnosis of acute exacerbation of chronic osteomyelitis triggered by aggravation of T2DM.

It is known that patients with T2DM have an increased susceptibility to various kinds of infection including diabetic foot osteomyelitis, especially when glycemic control is poor [5, 6]. In addition, in classification of osteomyelitis by cause, T2DM is one of most common conditions associated with osteomyelitis. In particular, under poorly controlled diabetic conditions, diabetic foot osteomyelitis could lead to sepsis [8] or a rare infectious disease such an intracardiac abscess [9]. On the other hand, there is the traditional classification for osteomyelitis, which is based on the duration and mechanism of infection [1]. In this classification, MRI is very useful for diagnosis and observation of subacute or chronic osteomyelitis. In this case, our patient had no ulcer or injury on the surface of his skin, but he had an abscess and inflammatory change in his right lower leg on MRI. He repeatedly experienced leg pain especially when glycemic control became very poor. Therefore, we considered that acute exacerbation of chronic osteomyelitis was triggered by aggravation of T2DM.

Patients with T2DM may develop osteomyelitis in their feet especially when they have foot ulcers [7]. It is thought that in such cases patients need long-term antibiotics therapy even when they have no symptoms [4]. Therefore, although this patient did not have foot ulcers or injury, we decided to do long-term antibiotics therapy, just in case, even after his symptoms disappeared.

It is known that recurrent flare up of chronic infection can occur at the same anatomical location. From the circumstantial evidence in this patient, we cannot completely exclude the possibility that there remained very small but chronic infection for a long period of time. We think, however, it is quite unlikely that there remained chronic infection for as long as 50 years or 8 years. Therefore, we think that it is likely that chronic infection was once substantially resolved and that poor glycemic control caused chronic osteomyelitis at the same position.

Taken together, this present patient suffered from acute osteomyelitis in his right lower leg when he was a baby. He then had acute exacerbation of chronic osteomyelitis when his glycemic control became very poor, although there was no ulcer or injury on his skin surface. We should be aware of the possibility of acute exacerbation of chronic osteomyelitis under immunotolerance status, such as poorly controlled T2DM, especially when a patient has a past history of acute osteomyelitis.

There is a limitation to this case report. We think that his chronic osteomyelitis was associated with acute osteomyelitis in his babyhood, because it was observed at the same position in his right lower leg. We cannot exclude the possibility that current osteomyelitis was newly caused by some other reason which was independent of previous osteomyelitis. It is well known, however, that patients with T2DM are immunocompromised hosts, especially when they are under poor glycemic control. Therefore, we think that acute exacerbation of chronic osteomyelitis was associated with acute osteomyelitis in his babyhood and that his repeated chronic osteomyelitis was triggered by aggravation of T2DM.

## Conclusions

We should bear in mind that T2DM is one of the major risk factors for osteomyelitis and that chronic osteomyelitis could be triggered by a disturbance in the glycemic control of patients with T2DM. In addition, when a patient has a past history of acute osteomyelitis, we should pay attention to the possible risk of acute exacerbation of chronic osteomyelitis especially under immunotolerance status, such as poorly controlled T2DM.

## References

[1] Lew DP, Waldvogel FA. Osteomyelitis. N Engl J Med. 1997;336:999–1007. 10.1056/NEJM199704033361406 9077380

[2] Peltola H, Pääkkönen M. Acute osteomyelitis in children. N Engl J Med. 2014;370:352–60. 10.1056/NEJMra1213956 24450893

[3] Lew DP, Waldvogel FA. Osteomyelitis. Lancet. 2004;364:369–79. 10.1016/S0140-6736(04)16727-5 15276398

[4] Tone A, Nguyen S, Devemy F, Topolinski H, Valette M, Cazaubiel M, *et al.* Six-week versus twelve-week antibiotic therapy for nonsurgically treated diabetic foot osteomyelitis: a multicenter open-label controlled randomized study. Diabetes Care. 2015;38:302–7. 10.2337/dc14-1514 25414157

[5] Shah BR, Hux JE. Quantifying the risk of infectious diseases for people with diabetes. Diabetes Care. 2003;26:510–3. 10.2337/diacare.26.2.510 12547890

[6] Benfield T, Jensen JS, Nordestgaard BG. Influence of diabetes and hyperglycaemia on infectious disease hospitalisation and outcome. Diabetologia. 2007;50:549–54. 10.1007/s00125-006-0570-3 17187246

[7] Ertugrul BM, Savk O, Ozturk B, Cobanoglu M, Oncu S, Sakarya S. The diagnosis of diabetic foot osteomyelitis: examination findings and laboratory values. Med Sci Monit. 2009;15:CR307–12. 19478702

[8] Brennan MB, Hess TM, Bartle B, Cooper JM, Kang J, Huang ES, *et al.* Diabetic foot ulcer severity predicts mortality among veterans with type 2 diabetes. J Diabetes Complicat. 2017;31:556–61. 10.1016/j.jdiacomp.2016.11.020 PMC5328848 27993523

[9] Pfirman KS, Haile R. Intracardiac Abscess and Pacemaker Lead Infection Secondary to Hematogenous Dissemination of Methicillin-Sensitive *Staphylococcus Aureus* from a Prior Diabetic Foot Ulcer and Osteomyelitis. Am J Case Rep. 2018;19:224–8. 10.12659/AJCR.906227 PMC5842747 29491344

